# Sensors for Entertainment

**DOI:** 10.3390/s16071102

**Published:** 2016-07-15

**Authors:** Fabrizio Lamberti, Andrea Sanna, Jon Rokne

**Affiliations:** 1 Dipartimento di Automatica e Informatica, Politecnico di Torino, Corso Duca degli Abruzzi 24, Torino 10129, Italy; andrea.sanna@polito.it; 2 Department of Computer Science, University of Calgary, 2500 University Dr. N.W., Calgary, AB T2N 1N4, Canada; rokne@ucalgary.ca

**Keywords:** games, interactive entertainment, human-machine interaction, wearable sensors, mobile devices, inertial sensors, bio-feedback, vision systems, sensor networks, education, rehabilitation, training, virtual, augmented and mixed reality, body, head and hand tracking, gesture recognition

## Abstract

Sensors are becoming ubiquitous in all areas of science, technology, and society. In this Special Issue on “Sensors for Entertainment”, developments in progress and the current state of application scenarios for sensors in the field of entertainment is explored.

## 1. Introduction

New and currently emerging entertainment systems are to an ever-increasing degree dependent on the availability of sensors. For mobile entertainment, touch and multi-touch displays are the most common ways of interacting with smartphones and tablets, presently the ultimate solution for mobile entertainment. TVs and gaming consoles allow users to control the devices using facial expressions, as well as hand and body gestures. Image and inertial data are similarly used to design ever more complex virtual and augmented reality systems. In-car infotainment equipment integrates sensors and speech recognition technology to enable hands-free operation. In the near future, it is expected that many other kinds of sensing technologies will be exploited in a progressively larger set of entertainment applications. Examples of such applications are eye and gaze tracking, bio-signals interpretation, haptic feedback, etc.

This Special Issue aims to exemplify some of the challenges that are being faced in the development of sensor-based solutions in the area of entertainment, with the aim to provide researchers with relevant case studies and solicit further developments in this field.

## 2. Content

A total of 11 articles were published in this Special Issue, covering a variety of topics related to the considered domain. Four of the articles are extended versions of selected works originally presented at the 7th International Conference on Intelligent Technologies for Interactive Entertainment, INTETAIN 2015 [1], a conference co-sponsored by the journal.

Saenz-de-Urturi et al. [2] present a 3D exergame created to help senior citizens perform physical activities and adopt a correct posture. A body tracking depth camera is used to assess the posture of a user. This posture is then compared with a set of reference positions to detect possible deviations. This provides users of the game with a quantitative feedback, which is expected to help them maintain an independent and healthy life.

The health domain is also explored by Martín-Ruiz et al. [3]. Here, the aim is to present the design of four interactive rehabilitation games. These games can be used to exercise the facial muscles of children with Cerebral Palsy (CP), with the aim to improve the swallowing process as well as facial expressions and speech. The sensor used is again a depth camera, but in this case facial tracking is adopted, rather than body tracking.

Children are also considered in the paper by Valpreda and Zonda [4]. Their goal was to create an educational game to teach children about problems related to food waste. To this aim, mixed and virtual reality, and elements of the Internet of Things (IoT) are combined with a mobile game, and used to let children experiment with crop growing. Sensors are exploited to characterize the environment the plant is growing in. The plant is represented by an avatar in the mobile game. By taking care of the amount of light, water, and heating/cooling the plant receives, children are expected to further develop their sense of responsibility, respect, and awareness of the environment.

In Reference [5], Díaz and Portalés present the temporal evolution of a system named HybridPLAY. This system combines a network of wireless sensors and actuators (accelerometers, gyroscopes, infrared proximity sensors, LEDs, and buzzers) with a mobile app that can be incorporated in any urban environment transforming the mobile app into a game with an engaging scenario. The aim of the system is to help children develop their physical and socio-communicative abilities. Sensors record children’s movements as they play, e.g., on the swings, slides, etc. of a playground, and transform the movements into actions, such as walk, jump, etc., for the mobile game. 

Inertial sensors were also used by Yu et al. [6]. Their goal was to find the best way to position a sensor on the body of a professional skier to capture turn motions that could be used for monitoring performance and for designing proper routine training. Additionally, data from inertial sensors were compared with information provided by foot pressure sensors in order to decide which of the two proposed technologies was the most suitable for performance monitoring. 

Alavi et al. [7] present the design of a system based on multiple inertial sensors for human motion capture, which also extends to gesture recognition. A user study is carried out to assess the performance of different algorithms in recognizing six gestures by using five wireless sensors attached to a user’s arms and upper abdomen. Results obtained indicate that high accuracy and interaction classification speed can be obtained for simple gestures through the proposed system.

In Reference [8], Invitto et al. study the perception of affordances of 3D objects during user interaction through a hand motion tracking system in an augmented reality environment. Event-related potentials (ERP) measured during a user study are compared with those collected while working with real objects, in order to show possible limits of current interactive systems used in a variety of fields, including entertainment. Results obtained show significant differences in the attentional components. In particular, it is shown that the use of virtual interaction alters the perception of objects by users, probably due to an incomplete interaction of the human multi-sensory processing system with the 3D objects.

Roig-Maimó et al. [9] focus on user interaction with mobile devices by using the onboard camera as a pointing device. A game is created that can be controlled by head movements. A comprehensive study is carried out by letting users test the system “in the wild”, showing the feasibility of using camera-based interfaces for mobile entertainment in different contexts and by different kinds of people. 

User interaction is also tackled by Alletto et al. [10]. However, in this case a different perspective is adopted, referred to as “egocentric”, where the user is wearing the camera. Information captured by the camera is used to position the user in the surrounding environment, making it possible to create effective location-based applications. Specially, in this work the cultural heritage domain is tackled, with the creation of an application that provides users with cues about historical landmarks they are actually looking at.

Loyola Ortiz-Vigon Uriarte et al. [11] explore the field of human-computer interaction from a multi-modal point of view. Specifically, a multi-sensor architecture is developed to use bio-feedback as a human-computer interaction technique. The devised system is used to control a game involving driving cars in risky situations, by collecting data from a pulsometer, a respirometer, an electromyography (EMG) sensor, a galvanic skin resistor (GSR), an eye tracker, and a body tracking system.

Lastly, Chen et al. [12] focus on user localization in outdoor environments. Here, the goal is to fuse global positioning system (GPS), gravity, and vision-based data to improve, in terms of tracking stability and robustness, the registration of mobile devices within different virtual environments.

## 3. Conclusions

The richness and diverseness of the papers submitted to this Special Issue confirm the importance of sensors in the wide domain represented by entertainment. The hope is that the reported experiences will inspire active researchers in this field and will contribute to further development of the domain.
